# Clinical efficacy of HBOT(hyperbaric oxygen therapy) in the treatment of foot ulcers in elderly diabetic patient: our experience

**DOI:** 10.1186/1471-2482-13-S1-A26

**Published:** 2013-09-16

**Authors:** Luciano Grimaldi, Marco Ferretti, Stefano Reggio, Umberto Robustelli, Massimiliano Fabozzi, Bruno Amato, Michele Danzi

**Affiliations:** 1 Department of Specialized Surgery, Division of Gastrointestinal Surgery Rehabilitation of Election and Emergency. “Federico II” University, Naples, Italy; 2 Emergency and General Surgery “AORN Cardarelli” Naples, Italy; 3 Department of General Surgery . “U. Parini” Hospital, Aosta, Italy

## Background

The development of foot ulcers is a serious complication in elderly diabetic patients. Its treatment is based on the use of different techniques, but when they fail that often lead to limb amputation. The efficacy of treatment with HBOT in diabetic foot ulcer has been evaluated for more than 20 years, but its use has never become routine, its use is a reality that in recent years is increasingly consolidating, especially as an adjuvant to conventional therapies and the NPWT (Negative Pressure Wound Therapy ) and dermal substitutes. Many studies prove its validity, experimentally the beneficial effects consist in the improvement of tissue perfusion, inflammatory cytokines down-regulation, fibroblasts proliferation, edema reduction , angiogenesis promotion and collagen production, it is also proven by the years the favorable effect against infectious component of the lesion [1,2]. In particular, the hyperbaric oxygen increases the bactericidal activity and is particularly toxic to anaerobes [3]. In addition, many studies, including some meta-analyzes, documenting the positive role of HBOT in reducing the risk of amputation, although a recent meta-analysis it is clear the short-term benefit, but for the long-term studies would be needed to be so designated such as to minimize any bias [4][5].

In our center, we evaluated the use of hyperbaric oxygen therapy to reduce limb amputation risk.

## Methods

This observational study, open-label short-term evaluated the clinical efficacy of this method integrated medical therapy, treatment of wound cleansing and debridement, and topical applications of hydrocolloids, alginates, polyacrylates and foams. After obtaining informed consents, 7 diabetic patients with foot ulcers (n = 7) were enrolled and treated with cycles of hyperbaric oxygen therapy (2.4 ATA) for a period of about 85 minutes for 5 days a week, during the period between January and September 2012, with follow-up until May 2013.Among the exclusion criteria we considered: patients with compromised bone and / or Charcot’s foot, Wagner class greater than III, non elegible patients for HBOT (AP chest X-ray, visit ENT, cardiological examination with ECG, laboratory tests to assess feasibility), then patients with severe impairment of arterial district in the affected arm. All patients have done foot X-ray and ECO Color Doppler of lower limb as preliminary study, which showed: four patients Wagner class II, Winsor index between 0.9 and 1, three patients Wagner Class III, with winsor pathological index. The treatment was carried out in two stages: at first, the patients were treated with medical therapy, surgical debridement, exudate management and stimulation of granulation and epithelialization with advanced wound dressings, wound swabs and orthotics , in a second time were matched HBOT cycles. The follow-up was done by clinical and biochemical controls with particular attention to the glycemic profile and obtaining optimal levels of glycated hemoglobin, and taking cilostazol tablets 100 mg, possibly associated with antiplatelets (cardioaspirin, clopidogrel). We established two outcome measures : 1) surgical healing of the lesions, 2) amputation (short-term assessment by the end of treatment until May 2013).

## Results

The demographic characteristics of our analyzed sample : 5M, 2F, all type II diabetic patients, mean age 66 years. On the basis of our preliminary data, we observed that 3 patients needed just two weeks of HBOT, while other 2 ones needed 4 weeks to get the necessary surgical healing of the lesions, seen as complete epithelial regeneration, also evaluated with ultrasound of the foot to highlight the possible persistence of outbreaks internal abscess. Other 2 patients required 6 weeks of HBOT. There were no adverse events. After almost a year, no one has suffered amputation of the limb and in only one case it was observed ulcer recurrence, in January 2013 , treated with the same method, and healed in 6 weeks of treatment.

## Conclusions

The management and treatment of foot ulcers in diabetic patients often require months of medical and surgical therapy, but if HBOT is associated the healing process takes place in less time, and this may even reduce the cost for the National Health System, despite the HBOT cycles are expensive. Currently, therefore, we can say that a lot of data available from international studies show that hyperbaric oxygen is excellent and valuable support in the healing process in patients with ulcer formed, and even though preliminary results of our study confirm this trend. We have to say that further studies are needed to validate the results obtained in the long term, and that the risk of amputation breakdown in these patients is the result of an intensive follow-up with a focus on medical therapy for good glycemic control and a good cardiovascular prevention, the pathophysiological processes key that contribute to determine these pathological lesions.
